# Non-Isothermal Crystallization Kinetics of Poly(4-Hydroxybutyrate) Biopolymer

**DOI:** 10.3390/molecules24152840

**Published:** 2019-08-05

**Authors:** Ina Keridou, Luis J. del Valle, Lutz Funk, Pau Turon, Lourdes Franco, Jordi Puiggalí

**Affiliations:** 1 Departament d’Enginyeria Química, Universitat Politècnica de Catalunya, Escola d’Enginyeria de Barcelona Est-EEBE, c/Eduard Maristany 10-14, 08019 Barcelona, Spain; 2 Center for Research in Nano-Engineering, Universitat Politècnica de Catalunya, Campus Sud, Edifici C’, c/Pasqual i Vila s/n, E-08028 Barcelona, Spain; 3 B. Braun Surgical, S.A. Carretera de Terrassa 121, 08191 Rubí (Barcelona), Spain

**Keywords:** poly(4-hydroxybutyric acid), bioabsorbable sutures, crystallization kinetics, non-isothermal crystallization, isoconversional methods, spherulites, synchrotron radiation

## Abstract

The non-isothermal crystallization of the biodegradable poly(4-hydroxybutyrate) (P4HB) has been studied by means of differential scanning calorimetry (DSC) and polarizing optical microscopy (POM). In the first case, Avrami, Ozawa, Mo, Cazé, and Friedman methodologies were applied. The isoconversional approach developed by Vyazovkin allowed also the determination of a secondary nucleation parameter of 2.10 × 10^5^ K^2^ and estimating a temperature close to 10 °C for the maximum crystal growth rate. Similar values (i.e., 2.22 × 10^5^ K^2^ and 9 °C) were evaluated from non-isothermal Avrami parameters. All experimental data corresponded to a limited region where the polymer crystallized according to a single regime. Negative and ringed spherulites were always obtained from the non-isothermal crystallization of P4HB from the melt. The texture of spherulites was dependent on the crystallization temperature, and specifically, the interring spacing decreased with the decrease of the crystallization temperature (*T_c_*). Synchrotron data indicated that the thickness of the constitutive lamellae varied with the cooling rate, being deduced as a lamellar insertion mechanism that became more relevant when the cooling rate increased. POM non-isothermal measurements were also consistent with a single crystallization regime and provided direct measurements of the crystallization growth rate (*G*). Analysis of the POM data gave a secondary nucleation constant and a bell-shaped *G*-*T_c_* dependence that was in relative agreement with DSC analysis. All non-isothermal data were finally compared with information derived from previous isothermal analyses.

## 1. Introduction

Poly(4-hydroxybutyrate) (P4HB) is a biodegradable, linear, and aliphatic polyester with interesting applications in the biomedical field. This is mainly associated with its use as a wound closure material [1]. This hydroxyalkanoate (HA) derivative can easily be produced from microorganisms (e.g., *Escherichia coli K12*) by recombinant fermentation under a deficit of nutrients or other stress limitations [2,3,4]. Basically, the polymer is employed by microorganisms (as other polyhydroxyalkanoates (PHAs), such as poly(3-hydroxybutyrate) (P3HB) and polyhydroxyvalerate (PHV), as an energy storage form produced by the carbon assimilation from glucose or starch sources. Biosynthesis is the only practical pathway to produce P4HB, since samples with a reasonable molecular weight cannot be obtained from conventional chemical synthesis (e.g., ring-opening polymerization of butyrolactone) [5,6].

P4HB has exceptional mechanical properties which allow the preparation of strong fibers with good retention in vivo [7]. The use of P4HB for soft tissue ligation was approved by the food and drug administration (FDA) in 2007, being commercialized as a long-term absorbable monofilament suture under the trademark of MonoMax (B. Braun Surgical, S. A.). In fact, P4HB is the only PHA allowed for regulatory agencies (e.g., USFDA and EU) to be used in clinical applications [8,9], such as reconstructive surgery, materials for cardiovascular applications, such as vascular grafts and stents [10], and devices for repair of hernias [11], ligaments and tendons, are other well-known applications of P4HB [1].

Probably the most remarkable feature of P4HB is not only its high ductility and flexibility (i.e., the elongation at break can reach a value of 1000%) but also the great interest for the preparation of copolymers with other HAs. Thus, the highly brittle and crystalline P3HB can easily be modified by copolymerization with different molar fractions of the 4HB monomer to obtain a group of materials with suitable mechanical strength and properties, including degradation rate [12].

On the other hand, P4HB (-[O(CH_2_)_3_CO]_n_-) has different properties than the similar linear polyesters with a lower (i.e., polyglycolide, -[OCH_2_CO]_n_-, PGA) and higher (i.e., poly(ε-caprolactone), -[O(CH_2_)_5_CO]_n_-, PCL) number of carbon atoms in their chemical repeat unit. Surprisingly, a continuous evolution of properties with the length of the repeat unit is not observed even while maintaining its parity, that is avoiding great changes of the crystalline structure. For example, typical elongation at break values are <3%, 1000%, and 80%, while elastic modulus values are 6900 MPa, 70 Mpa, and 400 MPa for PGA, P4HB, and PCL, respectively [10]. Differences in the degradation rate are clear (e.g., fast and slow for PGA and P4HB, respectively), and it is of interest to explore the use of combinations of such materials to tune the final degradability of the material [13].

The peculiar properties of P4HB have attracted great attention for the development of new materials for applications in the biomedical field. However, research concerning physical characterization is surprisingly relatively scarce, especially considering that the above-indicated differences with related polyesters. To the best of our knowledge works concerning physical characterization of P4HB only include the study of its crystalline structure by both X-ray and electron diffraction techniques [14,15,16], morphologic studies [16], evaluation of hydrolytic and enzymatic degradation mechanisms [16,17], and determination of basic thermal and mechanical properties [1].

Thermal properties of P4HB are strongly dependent on the preparation conditions. Samples are crystalline and have shown a preferential melting peak at 72 °C after annealing at an appropriate temperature, under stress conditions or from solution crystallization. By contrast, a decrease in the melting temperature at 58 °C is characteristic when samples are crystallized after melting. Control of the crystallization process seems fundamental considering the relatively low melting point that becomes close to room temperature and the derived applications where crystallinity plays a significant role (i.e., degradation rate and even elastic behavior). Isothermal crystallization studies of P4HB have recently been carried out considering both calorimetric (DSC) and optical microscopy (OM) experimental data [18]. The obtained data covered a very narrow range of crystallization temperatures (i.e., 24–38 °C and 37–49 °C for DSC and OM observations, respectively) due to experimental limitations (e.g., high primary nucleation and slow crystallization rate at low and high temperatures, respectively). Results indicated a crystallization process from the melt state defined by an averaged Avrami exponent of 2.56. Crystallization occurred according to a single regime characterized by a secondary nucleation constant of 1.69 × 10^5^ K^2^ (from DSC data) and 1.58 × 10^5^ K^2^ (from OM data).

Nowadays, efforts are focused on understanding the non-isothermal crystallization behavior of semi crystalline polymers since this is more appropriate to describe the usual processing conditions and can even be useful in the description of the crystallization process for a wider temperature range. Different methodologies have been developed to carry out the evaluation of non-isothermal crystallization from DSC and OM experimental data. Results are in general controversial when different methods are compared, especially when interpretation and theoretical approximations are not clear. The goals of the present paper are a) the specific study of non-isothermal crystallization of P4HB, a polymer with increasing applied interest and with a crystallization process scarcely studied; b) a comparison of the non-isothermal process with isothermal crystallization data recently evaluated, and c) the evaluation of the more significant highlights given by the different theories derived from DSC and OM data.

## 2. Results and Discussion

### 2.1. Limitations of the Avrami Analysis of Non-Isothermal Crystallization of P4HB

**Figure 1:**
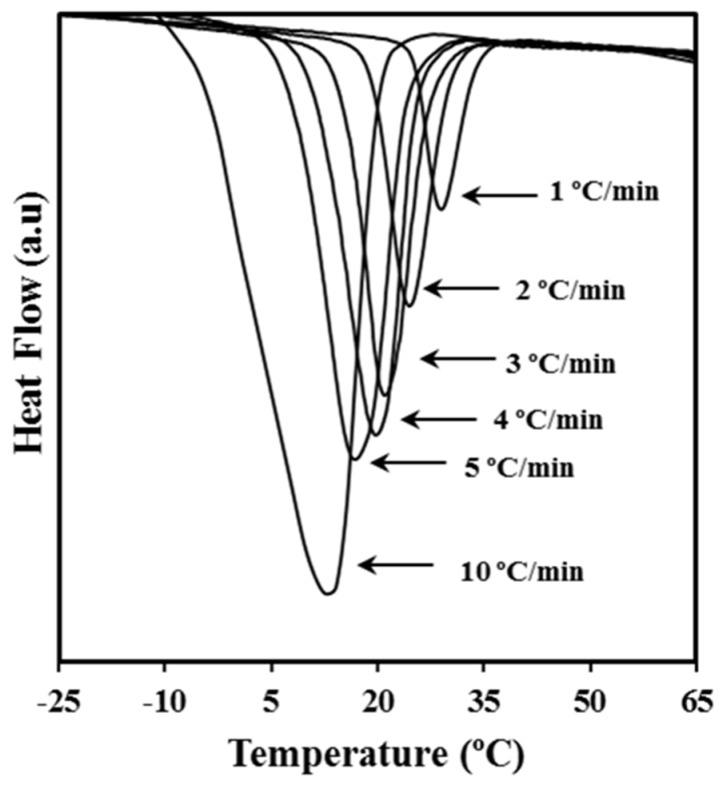
Dynamic differential scanning calorimetry (DSC) curves obtained at the indicated cooling rates for poly(4-hydroxybutyrate) (P4HB) crystallization from the melt state.

Figure 1 shows the dynamic DSC exotherms obtained by cooling melted P4HB samples at different rates. Logically, peaks moved progressively to lower temperatures as the cooling rate increased. Calorimetric data allowed the determination of the relative degree of crystallinity at any temperature, *χ* (*T*), for all cooling rates by the expression

(1) $$\chi \left(T\right)=\frac{\int_{T_{0}}^{T}\left({\mathrm{dH}}_{c}/\mathrm{dT}\right)\mathrm{dT}}{\int_{T_{0}}^{T_{\infty }}\left({\mathrm{dH}}_{c}/\mathrm{dT}\right)\mathrm{dT}}$$

where d*H_c_* is the enthalpy of crystallization released within an infinitesimal temperature range d*T*, *T_0_* denotes the initial crystallization temperature, and *T_∞_* is the temperature required to complete the crystallization process. Thus, the denominator corresponds to the overall enthalpy of crystallization for specific heating/cooling conditions. Note that this relative crystallinity is obviously higher than the real extent of crystallization, which is limited by the slow dynamics of polymeric molecular chains.

**Figure 2:**
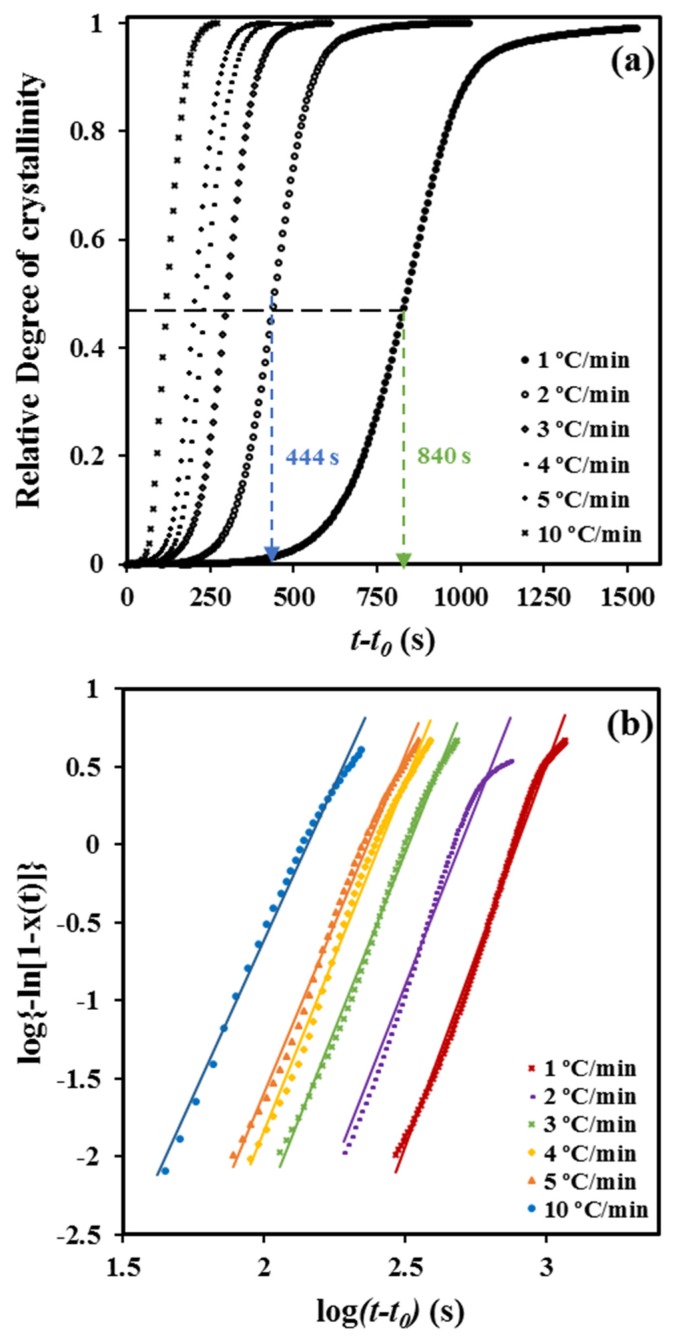
Time evolution of relative crystallinity (**a**) and Avrami plots, (**b**) at the indicated cooling rates for the non-isothermal crystallization of P4HB.

The time dependence of the degree of crystallinity (Figure 2a) can be derived considering the relationship

(*t* − *t_0_*) = (*T* − *T_0_*)/ *φ* (2)

where *T_0_* is the temperature when crystallization begins (*t* = *t_0_*) and *φ* is the cooling rate.

The typical Avrami analysis can be applied to these non-isothermal experiments based on Equation (3).

1 − *χ*(*t* − *t_0_*) = exp [−*Z* (*t* − *t_0_*)*^n^*], (3)

where *Z* is the temperature-dependent rate constant and *n* the Avrami exponent.

This exponent has a physical sense for isothermal crystallization of semi crystalline polymers despite being initially postulated for the study of the phase transformation of metals [19,20]. It has been established that the exponent varies according to the dimensionality of the crystal growth and the type of nucleation [21]. Namely, a time-dependent thermal nucleation (i.e., homogeneous nucleation and sporadic heterogeneous nucleation) can be differentiated from athermal nucleation (i.e., instantaneous heterogeneous nucleation) after evaluating the crystal dimensionality. Unfortunately, the direct application of the Avrami equation to the evaluation of non-isothermal crystallization merely corresponds to a mathematical fitting. This allows the evaluation of the variation of crystallinity with crystallization time, but parameters lose their physical meaning since, for example, values of the exponent become usually higher than four.

Plots of log {−ln [1 – χ (*t* − *t_0_*)]} versus log (*t* − *t_0_*) showed a good linearity (Figure 2b) before the start of the secondary crystallization process associated with the impingement of spherulitic crystals (i.e., the decrease of the dimensionality of the crystal growth). In fact, the linearity is observed, in our case, up to a relative crystallinity of 0.92.

Table 1 summarizes the main kinetic parameters deduced from the Avrami analysis, including the overall crystallization rate, *k*, calculated as *Z*^1/*n*^. This rate has units of s^−1^ and consequently, can be used to compare data from crystallizations having different Avrami exponents. Note that the usually employed *Z* parameter is not useful since it has units of s^−*n*^ (i.e., it is dependent on the change of nucleation mechanism and crystal growth dimensionality). Logically, the crystallization became faster as the cooling rate increased and specifically a change from 1.30 × 10^3^ to 4.40 × 10^3^ was detected when the rate was increased from 1 °C/min to 5 °C/min. The general observed trend was the decrease of the Avrami exponent (being 5.0 the average value) when the cooling rates were increasing. This is clearly higher than the postulated value for a maximum crystal dimensionality and a homogeneous (or even a sporadic heterogeneous) nucleation, making the deduction of the crystallization mechanism as above indicated impossible. Nevertheless, the observed decrease suggests that the dimensionality decreased as crystallization was conducted faster. Reported data for the non-isothermal crystallization of the related PCL polyester also gave a high Avrami exponent (i.e., *n* between 3 and 4), although it was interpreted as a three-dimensional spherulitic growth with homogeneous nucleation [22].

Table 1 also shows a satisfying agreement between the reciprocal crystallization half-times (1/*τ*_1/2_) that were directly determined from the experimental data and those that were deduced from the Avrami parameters (i.e., 1/*τ*_1/2 =_ (*Z*/ln2)^1/*n*^). The deduced parameters are at least appropriate to simulate the non-isothermal crystallization process.

### 2.2. Alternatives to the Avrami Analysis for the Non-Isothermal Crystallization of P4HB

Ozawa [23] proposed a modified Avrami equation that directly considers the effect of the cooling rate. The approach assumes that a non-isothermal process is the result of an infinite number of small isothermal steps. The Ozawa equation was formulated by applying the mathematical derivation of Evans [24] to the Avrami equation and considering a constant cooling rate.

1 − *χ* ( *T* ) = exp (−*R*( *T* )/*φ^m^* ) (4)

where *R*(*T*) is a cooling function that depends on the temperature of the process and *m* is the so-named Ozawa exponent. The difference of the exponent with the above indicated Avrami exponent is not clear, and generally, it has been interpreted in the same way [25].

**Figure 3:**
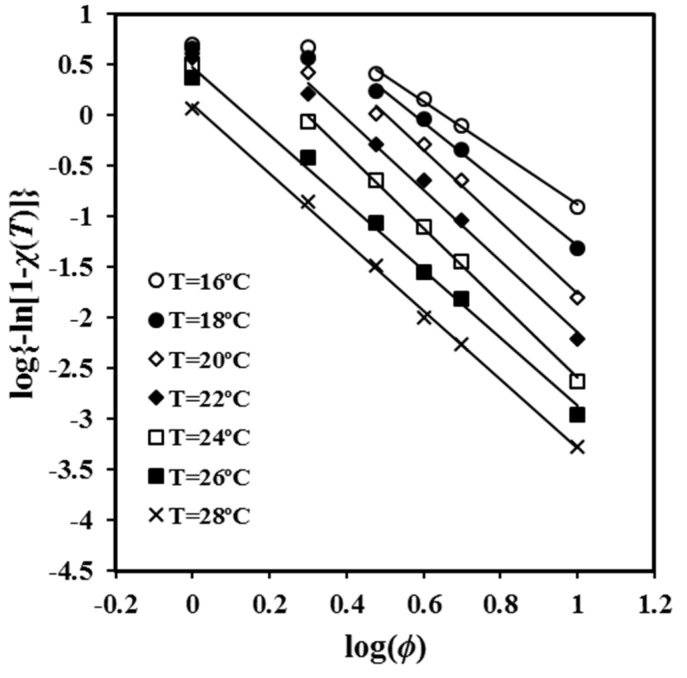
Plots of log{−ln [1 − *χ*(*T*)]} versus log*φ* for non-isothermal crystallizations of P4HB at the indicated temperatures.

The exponent can be deduced from the plot of log{−ln[1 − *χ*(*T*)]} versus log *φ* for conversions determined at the same temperature and different cooling rates. The main limitation of the method is that the linearity is observed for a restricted range of cooling rates, as can be observed in Figure 3. In fact, the absence of linearity becomes more evident as the process becomes faster (i.e., the temperature is lower). In other words, the analysis is highly sensitive to the variation between primary and secondary crystallization processes. It was noted in the previous section that secondary crystallization only becomes significant when the degree of crystallinity becomes very high. Thus, plots performed at high crystallization temperatures are linear over a wide range of cooling rates due to the slow crystallization and the difficulty of entering into the secondary crystallization region.

The slopes of the different straight segments shown in Figure 3 are close to −3.5, which suggests athermal nucleation and three-dimensional spherulitic growth. The value of the Avrami exponent is in clear contradiction with the reported values from isothermal studies, a feature that is congruent with the previous discussion and that points out the limitation of the application of the Avrami analysis to non-isothermal crystallization studies. The values of the exponent clearly decreased at low crystallization temperatures when cooling rates were low as a consequence of the increasing secondary crystallization. Thus, exponents of 1.44, 1.87, and 2.35 were determined in the cooling rate region between 2 and 3 °C/min for temperatures of 16 °C, 18 °C, and 20 °C, respectively. Low values of 1.14 and 1.87 were estimated in the cooling rate region between 1 and 2 °C/min for temperatures of 22 °C and 24 °C, respectively.

**Figure 4:**
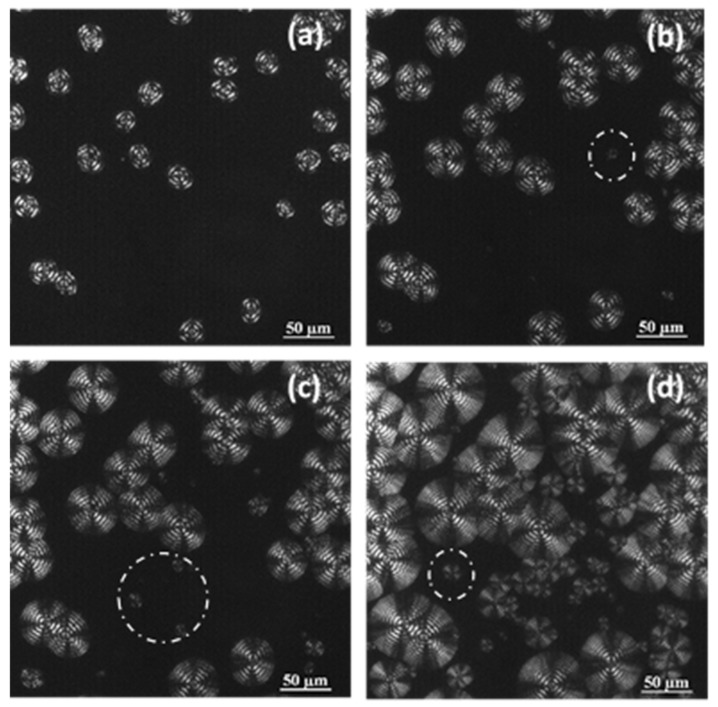
Optical micrographs of P4HB spherulites formed at the indicated non-isothermal crystallization times by cooling the sample at a rate of 0.5 °C/min from 45 °C, where some spherulites were isothermally grown (60 min) from the melt state. Micrographs were taken at temperatures of 44 °C (**a**), 38 °C (**b**), 36 °C (**c**), and 33 °C (**d**).

**Figure 5:**
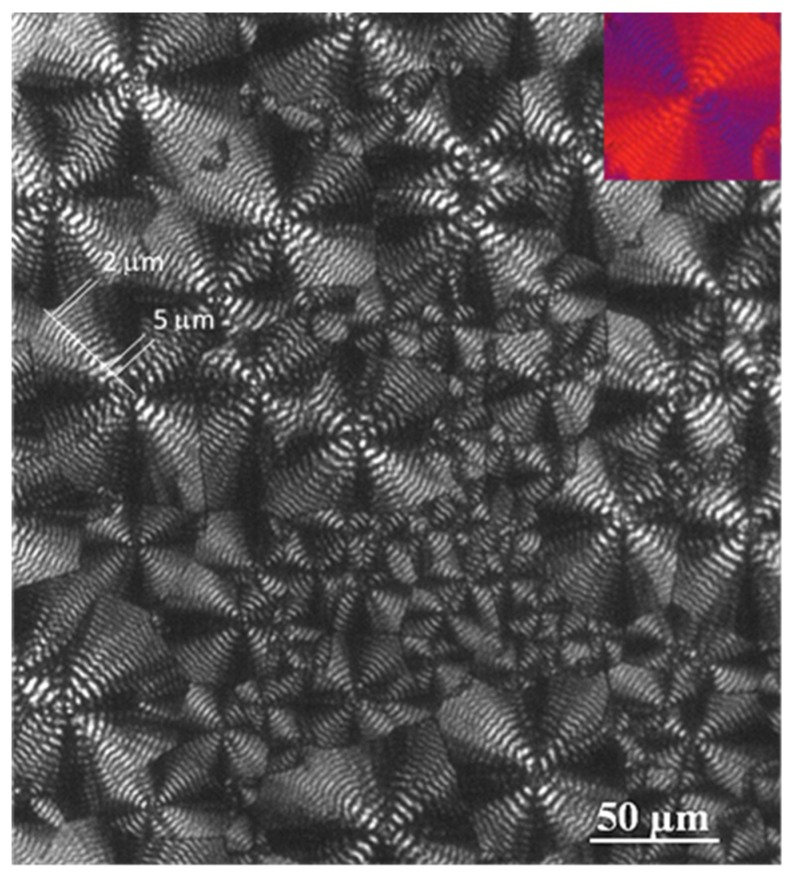
Optical micrograph at the end of the non-isothermal crystallization (0.5 °C/min) of P4HB. The decrease in the interring spacing during crystallization is evident. Inset shows a micrograph taken with a first-order red tint plate to determine the birefringence sign.

Optical micrographs (Figure 4 and Figure 5) taken during the non-isothermal crystallization clearly show the development of banded spherulites with a negative birefringence and increasing nucleation as a consequence of the temperature decrease. For instance, the dashed circles that are observed indicate the apparition of new nuclei and spherulites during the crystallization that was performed at a representative cooling rate of 0.5 °C/min. Obviously, these non-isothermal experiments cannot demonstrate that the crystallization takes place according to an athermal process (i.e., the apparition of new nuclei during crystallization at a given temperature), but this feature was corroborated in previous isothermal crystallization studies. The spherulitic texture was variable (e.g., the width of bands shown in Figure 5 continuously decreased during the crystallization that began at 45 °C and finished at 29 °C), since changes were expected between regions crystallized at high and low temperatures.

Liu et al. [26] postulated an alternative calorimetric analysis based on the combination of typical Avrami and Ozawa treatments. Equation (5) (also known as the Mo equation) was derived.

log *φ* = log *F*(*T*) − *a* log (*t* − *t_o_*) (5)

where *F*(*T*) is a new kinetic function, defined as [*χ* (*T*)/*Z* (*T*)]^1/*m*^, and *a* is the ratio between apparent Avrami and Ozawa exponents (*n*/*m*).

The main problem of the model is the non-clear physical sense of *F*(*T*), which was defined as the cooling rate that must be chosen at a unit crystallization time to reach a certain crystallinity. *F*(*T*) increases with increasing crystallinity, indicating that a higher cooling rate is required. The main interest of Liu analysis concerns the evaluation/quantification of how the modifications of a system (e.g., incorporation of additives) may be reflected in a more difficult crystallization (i.e., higher *F* (*T* ) values for a given crystallinity).

**Figure 6:**
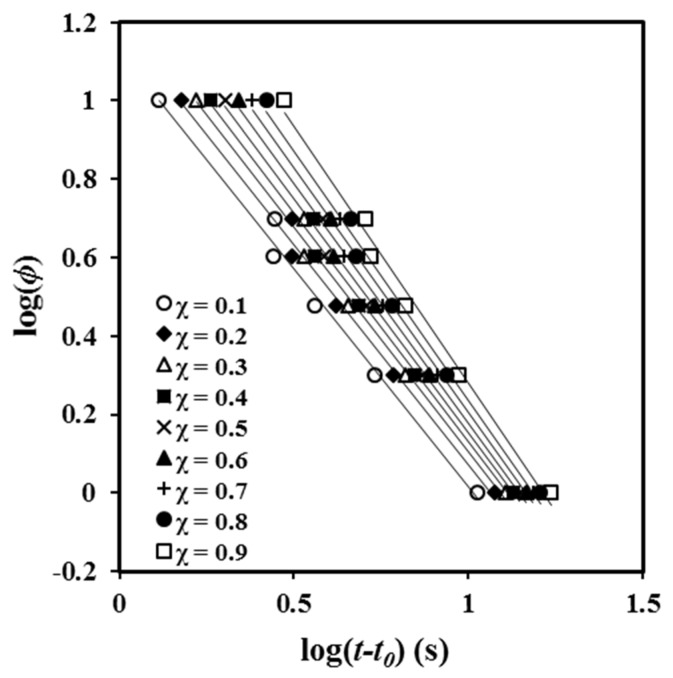
Plots of log *φ* versus log*(t* − *t_0_*) at the indicated crystallinities for non-isothermal crystallization of P4HB.

A plot of log *φ* versus log (*t* − *t_0_*) yields a series of straight lines (Figure 6) that suggest the validity of the Mo equation for the P4HB system. The intercept and slope of these lines can be used to estimate the kinematic parameters. The values of *F* (*T*) (Table 2) increased with crystallinity, indicating that the motion of molecular chains became slower, making the formation of crystals more difficult.

The second piece of information derived from the Liu and Mo analysis concerns the *n*/*m* ratio, which theoretically should be equal to 1 if equivalence of exponents is assumed. Different non-isothermal studies revealed as presumably a good equivalence between both exponents [25,27,28,29].

Table 2 shows that the values of *a* were almost constant and close to 1 (i.e., between 1.11 and 1.31). Nevertheless, the *a* values slightly increased with crystallinity (i.e., an increased dissimilarity between Avrami and Ozawa exponents was observed when crystallinity increased). Specifically, Avrami exponent became regularly higher than the Ozawa exponent. Note that Figure 3 demonstrates that Ozawa exponent changed and dramatically decreased at high crystallinity as a consequence of the great contribution of secondary crystallization. Previous studies performed with the related PCL polyester indicated a ratio higher than 1 and specifically an increase from 1.41 to 1.65 for conversions varying from 0.2 and 0.8 [22].

Cazé has also developed a methodology able to render an average value of the Avrami exponent for all the crystallization process [30]. The method hypothesizes that crystallization exotherms follow a Gaussian curve and considers three temperature inflection points: the onset temperature, the peak temperature, and the end crystallization temperature. The approach assumes that these three temperatures vary linearly with the cooling rate. A theoretical peak temperature *T′_p_* and a new constant *a′* can be estimated, assuming the following equation:

ln [1 − ln (1 − χ (*T*))] = *a’* (*T* − *T’_p_*) (6)

**Figure 7:**
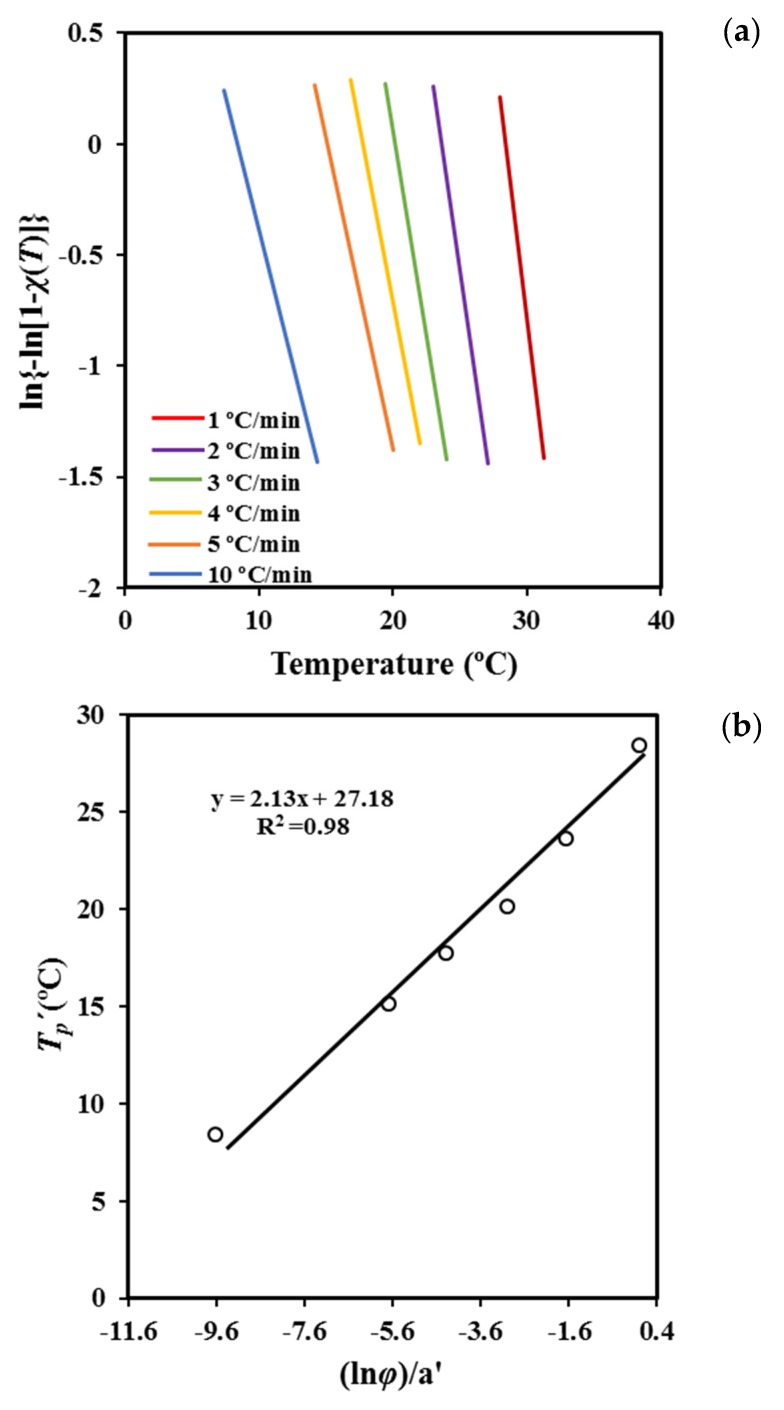
Plots of ln{−ln[1 − *χ*(*T*)]} against temperature for non-isothermal crystallization of P4HB with cooling rates as a parameter (**a**). Determination of the Avrami exponent using linear plots of *T_p_*′ against (ln *φ*)/a′ (**b**).

Plots of ln [1 − ln (1 − χ (T))] versus *T* at different cooling rates (Figure 7a) are linear and allow the indicated parameters (i.e., *a′* and *T′_p_*) to be calculated (Table 3). It is worth noting that Equation (6) is confined to the primary crystallization regime. The range of crystallinities starts at 2% to ensure precision and cover data in such a way that the correlation coefficient is greater than 0.99. Table 3 also shows a favorable agreement between the peak temperature that was estimated assuming the Cazé model and that directly determined from the experimental DSC data.

The deduced peak temperatures can then be related to the cooling rate (Figure 7b) by the expression

*T’_p_* = (*m*/*a’*) *ln φ* − *b’/a’* (7)

where *b’* is a new constant.

The plot of *T’_p_* versus ln *φ a’* (Figure 7b) gives straight lines with a slope equal to the estimated Ozawa exponent *m*. The values obtained for P4HB are close to 2.13, which has physical meaning and appears very close to those deduced from the isothermal analysis [18] (i.e., exponents varied between 2.35 and 2.62, with 2.56 being the average value). Note that the derived value of *m* corresponds to an Avrami exponent of 2.57 to 2.87 if the *n/m* ratio deduced from the Liu model is applied. The obtained results support the suitability of the Cazé methodology, as previously reported in the non-isothermal study of different polymers [31].

### 2.3. Isoconversional Methods. Activation Energy

Evaluation of the activation energy of a non-isothermal crystallization from the melt was performed by using the isoconversional method of Friedman [32]. This considers that the energy barrier of crystallization can vary during the non-isothermal melt crystallization according to Equation (8).

d*χ*(*T*)/d*t* = *A* exp (−Δ*E*/*RT*) *f*[*χ*(*T*)] (8)

where *A* is a preexponential factor, and *f* [*χ* (*T*)] is the crystallization model. This method assumes that the activation energy is only constant at a given extent of conversion and for the narrow temperature region associated with this conversion.

In fact, the temperature-dependent activation energy is a consequence of the non-Arrhenius behavior expected for the crystallization process. In this sense, it should be indicated that a mistake is derived when other simpler isoconversional methods, such as Kissinger [33], Kissinger–Akahira–Sunose [34], Ozawa [35], and Flynn and Wall [36], are applied. These methods were also problematic, as indicated by Vyazovkin for crystallization processes that are defined by cooling rate values [25,37].

**Figure 8:**
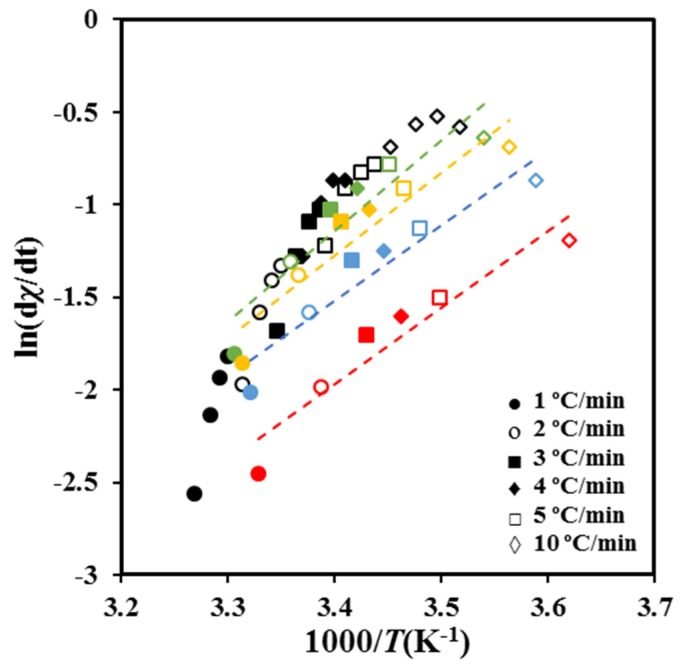
Plots of ln(d*χ*/dt)*_χ_* versus 1/*T* for non-isothermal crystallization of P4HB at the indicated cooling rates. Data corresponding to different relative degrees of crystallinity are represented using symbols and lines of different colors (i.e., green, yellow, blue, and red correspond to 0.5, 0.6, 0.7, and 0.8 values, respectively).

Crystallization experiments performed at different cooling rates allow obtaining values for ln [d*χ*(*T*)/d*t* ] at different temperatures and crystallization degrees. For a given conversion, the slopes of the linear plots of ln [d*χ*(*T*)/d*t* ] versus 1/*T* (Figure 8) determines Δ*E.*

**Figure 9:**
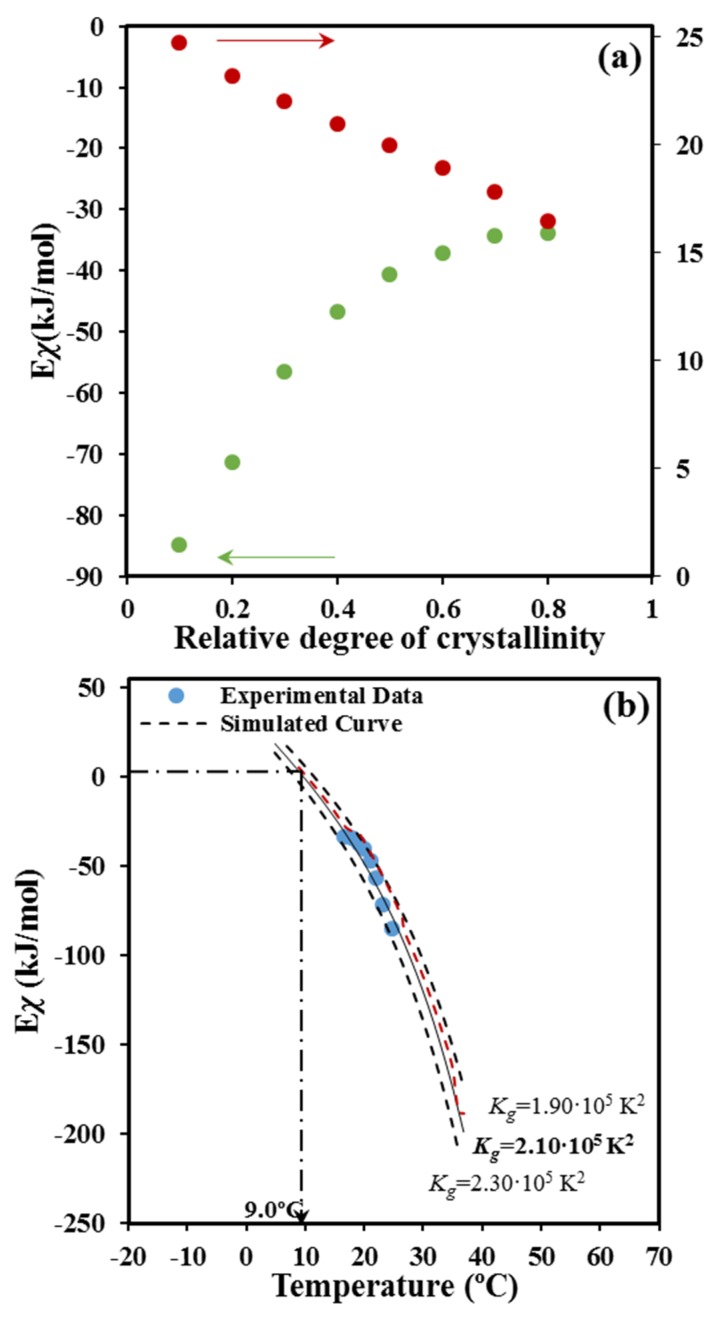
(**a**) Dependence of the activation energy of crystallization (●) and the average crystallization temperature (●) on crystallinity. (**b**) Experimental *Ex* vs. *T* data for non-isothermal crystallization of P4HB. The solid black line corresponds to the data calculated by Equation (11) and the optimized crystallization parameters. Arrow indicates the expected temperature for the maximum crystallization rate (i.e., the effective activation energy becomes equal to zero).

As shown in Figure 9a, the deduced values of the activation energy are negative as expected in the temperature range from the melting point down to the temperature of the maximum crystallization rate. The energy sign indicates that crystallization rates increase with decreasing crystallization temperatures. It should be pointed out that the effective activation energy varies between −45 kJ/mol and −98 kJ/mol covering a wide range of energies. Published results concerning PCL showed a similar variation with a change from −49 kJ mol^−1^ to −110 kJ mol^−1^ with χt the ranging from 10 to 90% [38].

Finally, the activation energy can be correlated (Figure 9b) with the crystallization temperature by considering the average temperature associated with a given degree of crystallinity (Figure 9a). Note that the estimation of the activation energy is based on the application of Arrhenius equation within small temperature regions associated with given values of the degree of crystallinity. The plot clearly shows that the activation energy was negative at temperatures higher than that associated to the maximum crystallization rate and tended to zero when temperature decreased, as extensively discussed by Vyazovkin and Dranca [39].

### 2.4. Secondary Nucleation Constant from Non-Isothermal Crystallization

DSC calorimetric data from non-isothermal crystallization experiments can also be employed to known crystal growth parameters as, for example, the secondary nucleation constant.

The Lauritzen–Hoffman model [40] is usually accepted to determine the crystal growth rate, *G*. According to this theory this rate is defined by two terms: a) The transport activation energy, U^*^, which expresses the difficulty of crystallizing segments to move across the liquid–crystal interface, and b) the secondary nucleation constant, *K_g_*, which evaluates the formation of new particles in the presence of an established population of previously formed particles. An increase of *K_g_* indicates a greater difficulty for the surface of a growing lamellar crystal to act as an effective nucleus.

The Lauritzen–Hoffman equation is defined by

*G* = *G_0_* exp [−U^*/^(*R* (*T_c_* − *T_∞_*))] × exp [−*K_g_*/(*T_c_* (Δ*T*) *f*)] (9)

where *G*_0_ is a constant preexponential factor, *T*_∞_ is the temperature below which molecular motion ceases, *T*_c_ is the selected crystallization temperature, *R* is the gas constant, Δ*T* is the degree of supercooling measured as the difference between the equilibrium melting temperature (*T*_m_^0^) and *T_c_* (i.e., Δ*T* = *T*_m_^0^ − *T*_c_), and *f* is a correction factor accounting for the variation in the bulk melting enthalpy per unit volume with temperature (*f* = 2*T*_c_/(*T*_m_^0^ + *T*_c_)).

The temperature dependence of *G* follows a bell-shaped curve due to the two exponential terms of Equation (9). In general, crystallizations from the melt takes place at relatively low degrees of supercooling (right region of the curve) where the influence of the transport term is not highly relevant. In this case, it is usual to perform calculations with standard U^*^ and *T*_∞_ values as those reported by Suzuki and Kovacs [41] (i.e., U^*^ = 1500 cal/mol and *T_∞_* = *T_g_ −* 30 K).

Hoffman and Lauritzen parameters can also be derived from non-isothermal crystallizations by using an isoconversional approach developed by Vyazovkin et al. [42] that has been satisfactorily tested for different polymers, such as poly(ethylene terephthalate) [42], poly(butylene naphthalate) [43], and poly(ethylene naphthalate) [39].

This isoconversional method is based on an explicit dependence of the activation energy on temperature (Equation (8)) that was derived assuming an equivalence of the temperature coefficients of the growth rate and the heat flow [44] (Equation (9)).

Δ*E_χ_* = [U*T^2^/(*T* − *T_∞_***)**^2^ ] + [*K_g_R*((*T_m_^0^*)^2^ − *T*^2^ − T*_m_^0^T*)/((*T_m_^0^* − T)^2^*T*)] (10)

d(ln *φ*)/*T* = d(ln *G*)/*T* (11)

The experimental temperature dependence of the activation energy that was determined in the previous section can be related to the theoretical one calculated from the right side of Equation (8). U^*^ and *K_g_* parameters are selected to get the best fit between theoretical and experimental data, which is the process simplified when standard U^*^ values can be employed. *T_m_^0^* and *T_g_* were taken equal to 79.9 and −45.4 °C, as previously evaluated from the calorimetric analysis of P4HB [18].

Figure 9b shows that a reasonable fit between experimental and predicted values is attained with the set of parameters: U* = 1500 cal/mol, *T_∞_* = *T_g_* − 30 K and *K_g_* = 2.10 × 10^5^ K^2^. For the sake of completeness simulated curves for *K_g_* values of 2.30 × 10^5^ K^2^ and 1.90 × 10^5^ K^2^ are also plotted (dashed lines), illustrating the impact caused by small changes in the nucleation parameter. In the same way, the non-significant influence caused by a change in U^*^ is also shown by the red dashed line calculated for U* = 1800 cal/mol, *T_∞_* = *T_g_* − 30 K, and *K_g_* = 2.10 × 10^5^ K^2^. Interestingly, the deduced parameters from the non-isothermal DSC data became very close to those evaluated from isothermal DSC experiments and direct OM measurements on the spherulitic growth. In this case values of the selected set of parameters became U* = 1500 cal/mol, *T_∞_* = *T_g_* − 30 K, and *K_g_* = 1.69 × 10^5^ K^2^ (DSC) and 1.58 × 10^5^ K^2^ (OM), which appear in acceptable agreement with those determined by the isoconversional methodology.

Figure 9b also shows that the activation energy becomes zero at a temperature of 9.0 °C. This zero of energy is associated with the maximum crystallization rate and therefore, should correspond to the maximum of the bell-shaped *G* − *T* curve. Note that at higher temperatures (i.e., the region dominated by the secondary nucleation) the activation energy becomes negative and progressively increases with decreasing the temperature. This feature means that the crystallization rate is enhanced with decreasing temperatures as discussed at length by Vyazovkin and Dranca [39]. At lower temperatures than those corresponding to the maximum rate, the activation energy becomes positive, indicating that *G* decreases when crystallization temperature decreases. Results of the non-isothermal study show an impressive agreement with the maximum growth rate determined from isothermal measurements from both optical microscopy (i.e., 15.0 °C) and even the calorimetric data (i.e., 14.0 °C) [18].

Overall, crystallization rates determined from DSC data and applying the Avrami analysis (Table 1) can be employed to determine the Lauritzen and Hoffman parameters, considering a proportionality between *k* and *G* values. In this case, Equation (12) can be applied.

*k* = *k_0_* exp [−U^*^/(*R* (*T_c_* − *T_∞_*))] × exp [−*K_g_*/(*T_c_* (Δ*T*) *f*)]. (12)

In addition, the temperature associated with each *k* value was taken as a rough approximation of the peak temperature determined for the DSC runs performed at the corresponding cooling rates.

**Figure 10:**
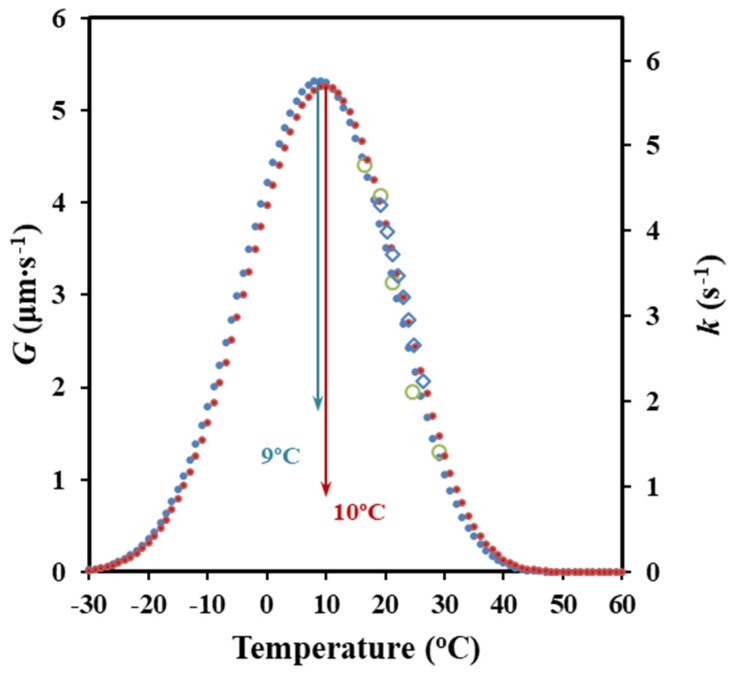
Comparison between bell-shaped curves of *G*/*k* temperature dependence obtained from DSC data and the two non-isothermal methodologies (from Avrami analysis (●) and activation energy (●) with their respective experimental results (o) (◊). An arbitrary value of *G_0_* has been selected to fit the maximum rate of both curves.

The plot of ln *k* + U^*^/(*R* (*T_c_* − *T_∞_*)) versus 1/[*T_c_* (Δ*T*) *f*)] gave a straight line with a slope (i.e., the *K_g_* value) of 2.22 × 10^5^ K^2^. It is very interesting to point out the great agreement with the secondary nucleation constant determined from the evaluation of activation energies. The similarity between both analyses can also be observed in Figure 10, where simulated bell-shaped curves from both sets of data are plotted. The advantages of the isoconversional method are clear since additional information concerning energies are obtained and furthermore approximations related to temperature, associated with each cooling rate, avoided.

### 2.5. Non-Isothermal Crystallization Studies by Optical Microscopy

Spherulitic growth rates for non-isothermal crystallizations can also be determined by optical microscopy [45,46,47]. In this case the evolution of the spherulite radius (*R*) with temperature (*T*) is followed for a constant cooling rate (d*T*/d*t*) [45,46]. Specifically, the growth rate is given by Equation (13). It is necessary to select an appropriate cooling rate to get the maximum information concerning the *G* − *T_c_* curve. Furthermore, a set of cooling rates can be employed if necessary to extend the curve to higher or lower temperatures.

*G* = d*R*/d*t* = (d*R*/d*T*) × (d*T*/d*t*) (13)

Evolution of the radius versus crystallization temperature during each selected cooling run allows obtaining a plot which is then fitted to polynomial equations. The selected equation corresponds to the lower order that renders a good regression coefficient (*r*). Growing rates (d*R*/d*T*) are then calculated at each crystallization temperature from the first derivative function of the polynomial equation. Experimental problems lie in the choice of the cooling rate required to maximize the crystallization temperature range where radii can be well measured, making necessary, in some cases, the use of various rates.

**Figure 11:**
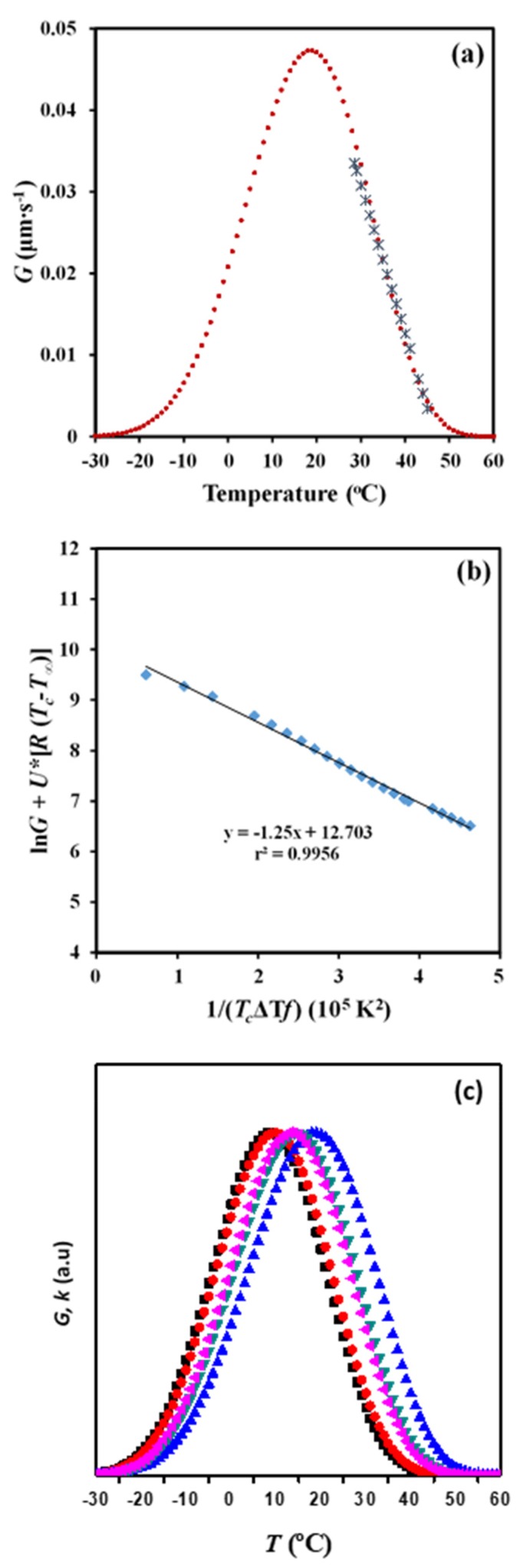
(**a**) Experimental and simulated dependence of the growth rate on crystallization temperature. (**b**) Plot of ln *G* + U*/*R* (*T_c_* − *T_∞_*) versus 1/*T_c_* (Δ*T*) *f* to determine the *K_g_* secondary nucleation parameter of P4HB. (**c**) Comparison between bell-shaped curves derived from non-isothermal DSC data ((●) from isoconversional and (■) from Avrami analyses), non-isothermal OM data (▲) and isothermal DSC data (◄) and OM (▼) data.

Figure 11a shows the evolution of the crystal growth rate of P4HB spherulites with crystallization temperature for a cooling rate of 0.5 °C/min. This rate allowed to cover a wide range of experimental data that are comparable with those available from non-isothermal DSC experiments. This range was also clearly higher than that defined by both DSC and POM isothermal crystallizations [18]. Therefore, kinetic analysis was carried out considering only the cooling rate of 0.5 °C/min. A second-order equation (*R* = 0.1091 *T*^2^ − 10.238 *T* + 239.89) gave a correlation coefficient of *r*^2^ = 0.996, which was slightly better than those calculated for higher-order equations.

Equation (7) can be applied to obtain crystallization parameters by considering the above deduced *G* – *T_c_* data. Thus, the representation of ln *G* + U^*^/(*R* (*T_c_* − *T_∞_*)) versus 1/[*T_c_* (Δ*T*) *f*)] gave a straight line with an intercept at the origin at ln *G_0_* and a negative slope equal to *K_g_* (Figure 11b). A single value of *K_g_* was observed in agreement with the above reported DSC data. This single value is a clear indication of a process that took place according to a single crystallization regime. The Lauritzen–Hoffman theory postulated the possibility of three regimes according to the type of nucleation on the crystal surface, being in some cases even related to different morphologies (e.g., axialites, banded/ringed spherulites, and non-ringed ones). Regime II is usually associated with ringed spherulites as those observed for P4HB in the considered temperature range. This regime II obeys to a nucleation rate on the crystal surface that is comparable or even greater than the lateral growth rate.

U* = 1500 cal/mol and *T_∞_* = *T_g_* − 30 K values gave a straight line with *r*^2^ = 0.995 and a *K_g_* parameter of 1.25 × 10^5^ K^2^. Note that this constant is in complete agreement with that determined by the isoconversional methodology and even the Avrami analysis. Nevertheless, the higher discrepancy was derived from this methodology. It seems that the method has a great advantage due to its simplicity, but some cautions must be taken into account concerning the precision of the derived results. It should be pointed out that OM analyses are independent of the nucleation rate and that some discrepancies with DSC measurements can also be justified.

It is also interesting to note that *K_g_* values determined from isothermal and non-isothermal crystallizations using DSC or POM data are close, with 1.7 ± 0.5 × 10^5^ K^2^ being the average value. In the same way, temperatures corresponding to the maximum of the bell-shaped curves that express the dependence of crystal growth rate or the overall crystallization rate on the crystallization temperature showed minimum deviations (i.e., 13 ± 5 °C).

Figure 11a compares the plots of the experimental and simulated *G* values versus crystallization temperature. The simulated curve was obtained by applying Equation (9) and the deduced Lauritzen–Hoffman parameters. This curve had a typical bell shape and showed a maximum at 19 °C, which was relatively higher than the temperature deduced from the isoconversional methodology and the DSC non-isothermal data.

Figure 11c compares the bell-shaped curves obtained from all the performed crystallization studies. For the sake of simplicity, arbitrary units have been employed for the ordinate axis due to the different represented rates (i.e., *G* and *k*). A relatively good agreement was in general observed between the isothermal and the non-isothermal studies and between DSC and POM techniques. In general, non-isothermal methods has advantages derived from their higher simplicity, the larger number of available experimental data and finally, the closer fit to realistic processing conditions. Crystallization under isothermal and non-isothermal conditions is obviously different and consequently, slight differences, such as those detected for the secondary nucleation constant and the temperature associated with the maximum crystallization rate, can be expected.

### 2.6. Synchrotron Data on Non-Isothermal Crystallization of P4HB

**Figure 12:**
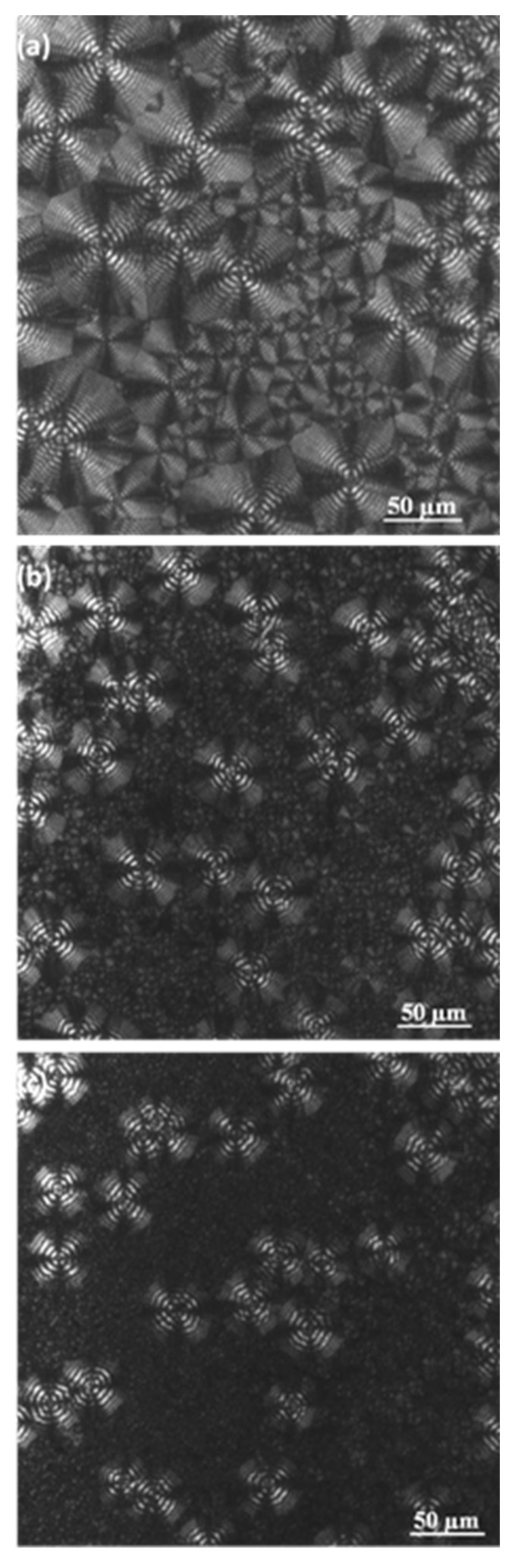
Optical micrographs taken at the end of the non-isothermal crystallizations of P4HB performed at cooling rates of 0.5 °C/min (**a**), 3 °C/min (**b**) and 5 °C/min (**c**).

Cooling rate has obviously an influence on the final morphology and even on the crystallinity of P4HB despite its rapid crystallization. Thus, melting enthalpy decreased from 39.6 kJ/mol to 35.2 kJ/mol when the cooling rate increased from 1 °C/min to 5 °C/min, while spherulitic size and texture changed (Figure 12). The average crystallization temperature decreased with the increase of the cooling rate (Figure 1) and consequently primary nucleation increased, leading to a decrease in the spherulite size (Figure 12). Spacing between rings was also temperature dependent as discussed earlier (Figure 5).

Morphology of constitutive lamellae depends not only on the crystallization rate, as indicated for the twisting period (i.e., interring spacing), but also on the lamellar thickness. Small-angle X-ray scattering patterns (SAXS) taken in real-time during the cooling process allowed to follow the evolution of lamellar thickness during crystallization and also allowed us to compare morphological parameters for different cooling rates through the use of the normalized correlation function:

(14) $$\gamma \left(r\right)=\int_{0}^{\infty }q^{2}I(q)\cos(qr)dq/\int_{0}^{\infty }q^{2}I(q)dq$$

where *I* (*q*) is the intensity of the SAXS peak at each value of the scattering vector (*q* = [4π/*λ*] sin *θ* = 2π/*d*, *θ* and *d* being the Bragg angle and the Bragg spacing, respectively).

Long period, *L_γ_*, amorphous layer thickness, *l_a_*, and crystalline lamellar thickness, *l_c_*_,_ can be determined by the normalized one-dimensional correlation function [48] and applying Vonk’s model [49] and Porod’s law to perform extrapolations to low and high *q* values.

**Figure 13:**
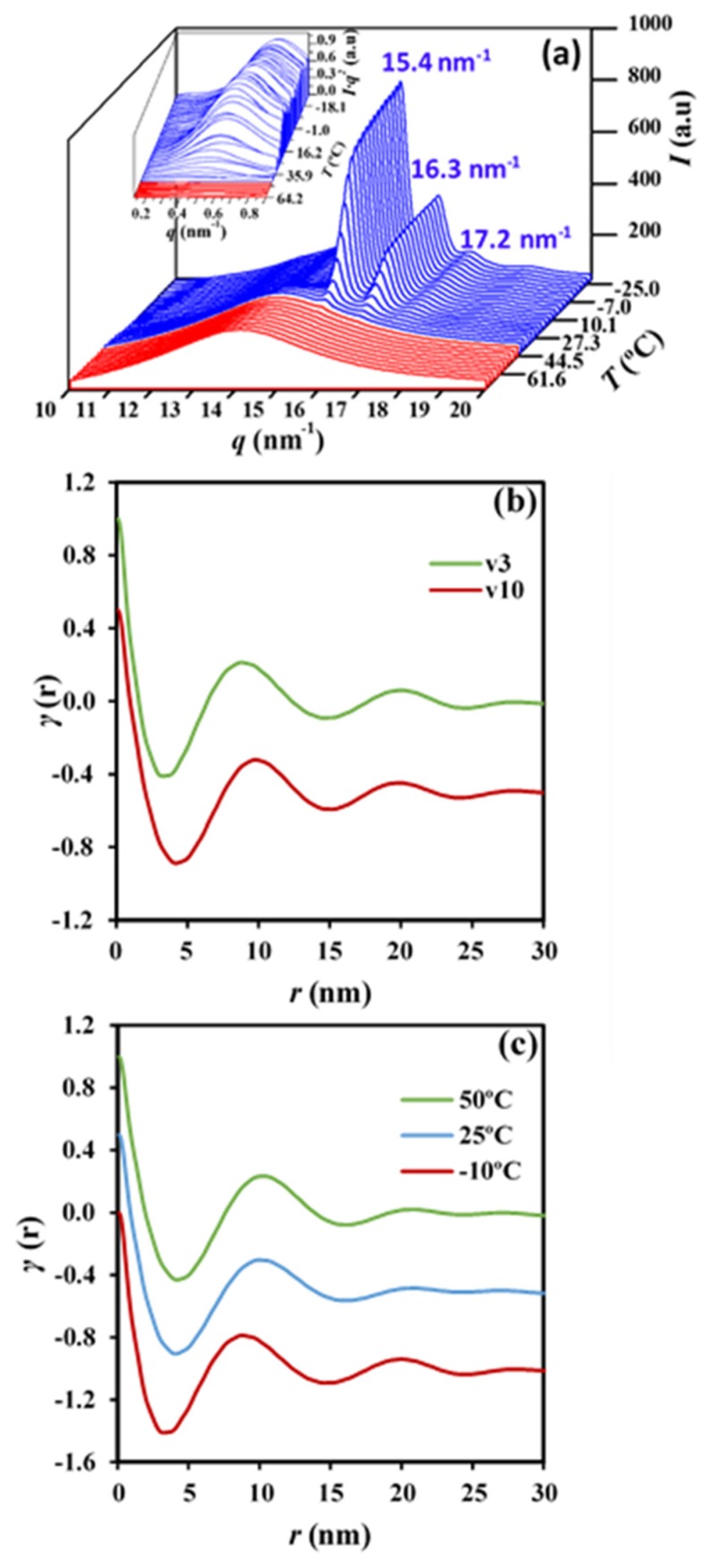
(**a**) Three-dimensional wide-angle X-ray diffraction (WAXD) and small-angle X-ray scattering patterns (SAXS) profiles of P4HB during cooling at a rate of 7 °C/min. (**b**) Correlation functions obtained after non-isothermal crystallizations of P4HB from the melt state performed at cooling rates of 3 and 10 °C/min. (**c**) SAXS correlation functions calculated at the indicated temperatures during the cooling run (3 °C/min) of a melted P4HB sample.

**Figure 14:**
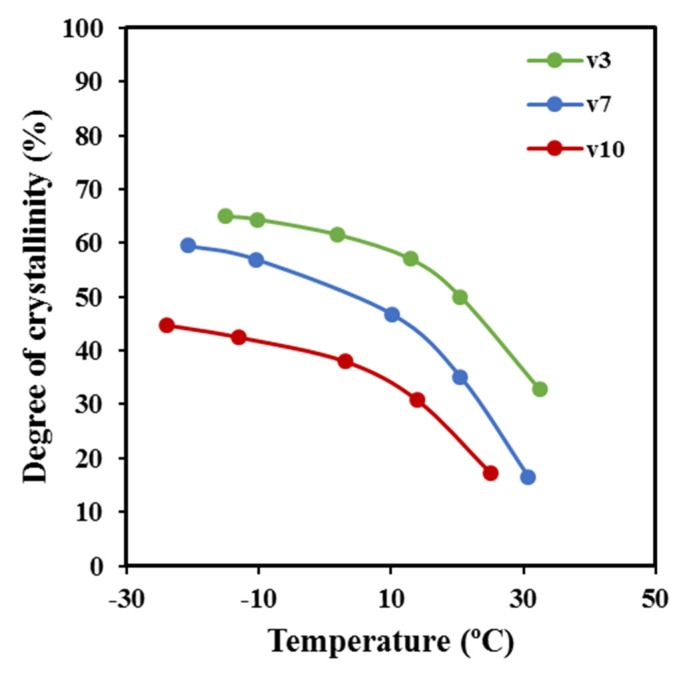
Evolution of the absolute degree of crystallinity determined from WAXD profiles with temperature for the indicated cooling rates.

Figure 13a shows the evolution of the SAXS peak during the non-isothermal crystallization from the melt at a representative cooling rate of 7 °C/min. This scattering peak appeared at the same temperature as the wide-angle X-ray reflections, which are presumable for a crystallization, where the supramolecular structure (lamellae) is developed at the same time that the molecular arrangement took place. Wide-angle X-ray diffraction (WAXD) profiles show two main peaks at 0.406 nm and 0.388 nm that correspond to the (110) and (200) reflections of the orthorhombic structure (*a* = 0.775 nm, *b* = 0.477 nm, and *c* (fiber axis) = 1.199 nm) reported for P4HB [15]. Deconvolution of WAXD profiles allowed for the determination of the temperature evolution of crystallinity during cooling runs performed at three different representative rates (Figure 14). Results clearly indicated that the increase of the cooling rate led to a decrease of the temperature at which crystallization started and as well as of the crystallinity (i.e., crystallinities around 65%, 60%, and 46% were determined for 3 °C/min, 7 °C/min, and 10 °C/min, respectively). Logically, the crystallization rate diminished when temperatures approached −20 °C (i.e., at some degrees above the glass transition temperature of −45.4 °C). It is also clear that samples are mainly crystallized at a lower average temperature when the cooling rate increased, and consequently, some influence on the derived lamellar morphology should be expected.

Figure 13b compares the correlation functions corresponding to the end of crystallizations performed at 10 °C/min and 3 °C/min. Slight but significant changes can be detected, with peaks being clearly defined for the crystallization performed at the lower rate. In this case, the contrast between the amorphous and crystalline regions is increased. Lamellae became narrower (8.70 nm versus 9.70 nm for *L_γ_*) for the crystallization performed at the lower rate, mainly as a consequence of the decrease in *l_c_* (i.e., 7.08 nm versus 7.61 nm), although a slight decrease in *l_a_* (i.e., 1.62 nm versus 2.09 nm) was also detected. Note that the average crystallization temperature is superior for samples crystallized at the lower cooling rate, consequently with an expected greater lamellar thickness. The opposite results that were attained can be explained due to a lamellar reinsertion mechanism which took place. This process is the consequence of the formation of thinner lamellar crystals between the loosely stacked primary lamellae and appears to be more significant when the crystallization process is slower. Figure 13c compares the correlation function obtained at the beginning, an intermediate stage, and the end of the crystallization process performed at a rate of 3 °C/min. It is clear that lamellar spacings decreased (i.e., 10.10/9.90/8.70 for *L_γ_*, 2.09/1.98/1.62 for *l_a_* and 8.01/7.92/7.08 for *l_c_*) when the temperature did. The indicated evolution (also observed for the other rates) points out a lamellar insertion mechanism and a molecular rearrangement in the amorphous layer. The bilayer model demonstrated an improvement in the molecular arrangement in the crystalline domains as visualized by the increase in the contrast of the electronic density of amorphous and crystalline layers, or even by the increase in the crystallinity within the lamellar stacks (*X_c_^SAXS^* = *l_c_*/*L_γ_*) which varied from 0.79 to 0.81.

## 3. Materials and Methods

### 3.1. Materials

Commercially available sutures of P4HB (Monomax^TM^, violet sample, USP 1) were kindly supplied by B. Braun Surgical S.A. The weight and number average of molecular weights of Monomax^TM^ samples were 215,000 and 68,000 g/mol, as determined by size exclusion chromatography (GPC).

### 3.2. Measurements

Molecular weight was estimated at room temperature by size exclusion chromatography (GPC) using a liquid chromatograph (model LC-8A, Shimadzu, Tokyo, Japan) equipped with an Empower computer program (Waters, Milford, MA, USA). A PL HFIP gel column (Polymer Lab, Agilent Technologies Deutschland GmbH, Böblingen, Germany) and a refractive index detector (Shimadzu RID-10A) were employed. The polymer was dissolved and eluted in 1,1,1,3,3,3-hexafluoroisopropanol containing CF_3_COONa (0.05 M) at a flow rate of 0.5 mL/min (injected volume 100 μL, sample concentration 2.0 mg/mL). The number and weight average of molecular weights were estimated using polymethyl methacrylate standards.

Calorimetric data were obtained by differential scanning calorimetry with a TA Instruments Q100 series with *T_zero_* technology and equipped with a refrigerated cooling system (RCS). Experiments were conducted under a flow of dry nitrogen with a sample weight of approximately 5 mg, and calibration was performed with indium. *T_zero_* calibration required two experiments: The first was performed without samples while sapphire disks were used in the second. Non-isothermal crystallization studies were performed by cooling previously molten samples (5 min at 100 °C) at rates varying from 5 to 1 °C/min.

The spherulitic growth rate was determined by optical microscopy using a Zeiss Axioskop 40 Pol light polarizing microscope equipped with a Linkam temperature control system configured by a THMS 600 heating and freezing stage connected to an LNP 94 liquid nitrogen cooling system. Spherulites were grown from homogeneous thin films prepared from the melt. Small sections of these films were pressed or smeared between two cover slides and inserted into the hot stage, producing samples with thicknesses close to 10 μm in all cases. Samples were kept at approximately 100 °C for 5 min to eliminate sample history effects. Then the sample was quickly cooled to 45 °C and led to isothermally grown for 60 min to generate enough nuclei for measurements and avoid the induction step. Subsequently, the radius of growing spherulites was monitored during crystallization with micrographs taken with a Zeiss AxiosCam MRC5 digital camera at appropriate time intervals. A first-order red tint plate was employed to determine the sign of spherulite birefringence under crossed polarizers.

Wide-angle X-ray diffraction (WAXD) and small-angle X-ray scattering patterns (SAXS) data were obtained at the NCD beamline (BL11) of the ALBA synchrotron facility (Cerdanyola del Vallès, Barcelona, Spain), by using a wavelength of 0.100 nm. A WAXD LX255-HS detector from Rayonix and an ImXPAD S1400 photon-counting detector were employed. Polymer samples were confined between Kapton films. WAXD and SAXS diffraction patterns were calibrated with Cr_2_O_3_ and silver behenate (AgBh), respectively. WAXD peaks were deconvoluted with the PeakFit v4 program by Jandel Scientific Software. The correlation function and corresponding parameters were calculated with the CORFUNC program for Fiber Diffraction/Non-Crystalline Diffraction provided by the Collaborative Computational Project 13.

## 4. Conclusions

Non-isothermal crystallization studies of P4HB were performed by calorimetric experiments and allowed the determination of crystallization parameters by considering both the classical Avrami analysis and isoconversional methods. In the first case, the determined Avrami parameters had no physical sense but gave an estimation of the variation of the overall crystallization rate with crystallization temperature, when average crystallization temperatures were assumed for experiments performed at different cooling rates. Additionally, this approximation made feasible the evaluation of the secondary nucleation constant. The isoconversional analysis provided information concerning the activation energy and allowed the estimation of both the temperature associated with the maximum crystallization rate and the secondary nucleation constant. Both methodologies were in remarkable agreement with the derived secondary nucleation constants (i.e., 2.10 × 10^5^ K^2^ and 2.22 × 10^5^ K^2^), as well as the temperatures associated with the maximum growth rate/overall crystallization rate (i.e., 9 and 10 °C). Optical microscopy data allowed the estimation of crystal growth rates at different crystallization temperatures. Results were independent of primary nucleation and in relatively good agreement with calorimetric analyses. Discrepancies could be associated with a lower precision of the method or to the effect caused by thermal nucleation. The obtained results are also comparable with previously reported data from isothermal crystallization. In this case, DSC and OM analysis were in a satisfactory agreement suggesting a scarce influence of thermal nucleation. The cooling rate had a great effect on the morphology and texture of spherulites, as well as the twisting and thickness of the constitutive lamellar crystals. WAXD synchrotron experiments allowed for the determination of final crystallinities and demonstrated a lower crystallization when the cooling rate increased. More interestingly, SAXS data indicated a lamellar insertion mechanism during crystallization that led to a decrease of the crystalline layer thickness. This process was more significant when the cooling rate decreased. The crystallinity of lamellar stacks slightly increased during the cooling run due to the similar thickness evolution of amorphous and crystalline layers.

## Figures and Tables

**Table 1:** Non-isothermal crystallization kinetic parameters deduced from differential scanning calorimetry (DSC) experiments for poly(4-hydroxybutyrate) (P4HB).

*Φ* (°C/min)	*n*	*Z* (s^−n^)	*k* × 10^3^ (s^−1^)	*τ_1/2_* (s)	(1/*τ_1/2_*) × 10^3^ (s^−1^)	(*Z*/ln2)^1/*n*^ × 10^3^ (s^−1^)
1	5.67	4.42 × 10^−17^	1.30	840	1.19	1.39
2	5.18	1.47 × 10^−14^	1.95	444	2.25	2.28
3	4.92	4.83 × 10^−13^	3.13	300	3.33	3.37
4	4.24	6.89 × 10^−11^	4.00	231	4.33	4.36
5	4.21	1.22 × 10^−10^	4.42	207	4.83	4.82
10	3.98	2.54 × 10^−9^	6.92	120	8.33	7.59

**Table 2:** Values of kinetic parameters at a given crystallinity estimated from the combined Liu model for non-isothermal crystallization of P4HB. ^1^

*χ (Τ)*	*α′*	*F (T)*	*r^2^*
0.1	−1.11	13.41	0.988
0.2	−1.12	15.70	0.990
0.3	−1.14	17.62	0.991
0.4	−1.16	19.74	0.991
0.5	−1.19	22.14	0.991
0.6	−1.22	24.85	0.990
0.7	−1.24	28.03	0.990
0.8	−1.28	32.30	0.989
0.9	−1.31	38.65	0.989

*^1^ r*: correlation factor.

**Table 3:** Characteristic crystallization parameters obtained for P4HB by using the methodology developed by Cazé et al. [30].

*φ* (°C/min)	*a* *′*	*T_p_′* (°C)	*T_p_^a^* (°C)
1	−0.49	28.43	28.92
2	−0.41	23.63	24.18
3	−0.37	20.15	20.86
4	−0.32	17.72	19.68
5	−0.28	15.14	16.87
10	−0.24	−9.61	13.27

^a^ Temperature determined for the exothermic peak observed in the cooling scans.
